# Upregulation of V-ATPase by STAT3 Activation Promotes Anoikis Resistance and Tumor Metastasis

**DOI:** 10.7150/jca.58670

**Published:** 2021-06-11

**Authors:** Funmilayo O. Adeshakin, Adeleye O. Adeshakin, Zhao Liu, Xiaoxu Lu, Jian Cheng, Pengchao Zhang, Dehong Yan, Guizhong Zhang, Xiaochun Wan

**Affiliations:** 1 Guangdong Immune Cell therapy Engineering and Technology Research Center, Center for Protein and Cell-based Drugs, Institute of Biomedicine and Biotechnology, Shenzhen Institute of Advanced Technology, Chinese Academy of Sciences, Shenzhen, 518055, China.; 2 University of Chinese Academy of Sciences, Beijing, 100864, China.; 3 School of Basic Medical Science, Jinzhou Medical University, Jinzhou, 121000, China.

**Keywords:** Anoikis, Metastasis, V-ATPase, STAT3, Misfolded protein accumulation

## Abstract

Most cancer mortality results from metastatic tumor cells and not the localized tumor. Overcoming anoikis is one of the most important steps for detached tumor cells to migrate and metastasize. However, the molecular mechanisms remain to be fully deciphered. Herein, our study revealed upregulation of vacuolar ATPase (V-ATPase) in cancer cells during ECM detachment plays a key role in anoikis evasion. V-ATPase is an enzyme complex that utilizes energy from ATP hydrolysis to maintain cellular homeostasis and had been reported to enhance cancer progression. In this study, V-ATPase inhibition sensitized human cervical cancer, breast cancer, and murine melanoma cells to anoikis via increased ROS production, accumulation of misfolded protein, and impaired pulmonary metastasis *in vivo*. Scavenging ROS restored anoikis resistance and clearance of misfolded protein accumulation in the tumor cells. Mechanistically, STAT3 upregulates V-ATPase expression while blockade of STAT3 activity repressed V-ATPase expression in these tumor cells as well as sensitized cells to anoikis, increased ROS production, and misfolded protein accumulation. Altogether, our data demonstrate an unreported role of STAT3 in mediating the upregulation of V-ATPase to promote anoikis resistance, thus provides an alternative option to target cancer metastasis.

## Introduction

Metastasis accounts for 90% of cancer deaths 1. Metastasis is a process by which localized tumor cells detach from the extracellular matrix (ECM), adopt a survival phenotype that enables them to circumvent anoikis and migrate to distal sites where they reseed to develop tumors 2. Anoikis is a natural program of cell clearance following cell detachment from its home in the ECM 3, it can either be via the intrinsic or extrinsic path like the apoptotic cascade of endonucleases activation, DNA damage and eventual cell death 4, 5. Compromised anoikis machinery is required for effectual metastasis of tumor cells to other sites. In other words, anoikis resistance is a precondition and a hallmark for the metastatic potential of tumor cells 3, 6. Targeting anoikis is a promising option for metastasis prevention but calls for a profound understanding of the mechanisms underlying anoikis resistance 6.

Alterations in reactive oxygen species (ROS) in tumor cells had been reported following cell detachment from the ECM 7-9. Both low and high ROS production were implicated to promote cancer 10, 11. However, studies reported elevated ROS impaired oncogenic transformation thus promoting cancer cell death 12, 13. Similarly, certain ROS regulatory genes were identified to either promote or prevent anoikis 11, 14-16. Therefore, how changes in ROS level in tumor cells contribute to anoikis remains to be fully understood. On the other hand, the molecular dynamics after tumor cells detach from the ECM contributing to anoikis abrogation and metastasis is yet to be fully demystified 6. To this effect, we cultured cervical cancer cells in culture dishes to demonstrate low adherence by pre-coating with poly 2-hydroxyethyl methacrylate (polyhema) to mimic ECM detachment. Our transcriptomic analysis revealed upregulation of Vacuolar ATPase (V-ATPase) genes compared to cells cultured in adherent condition.

Vacuolar ATPase is a multi-protein complex catalyzing ATP-dependent protons transport across intracellular and plasma membranes, its catalytic effect of proton transport brings about pH reduction in the cellular milieu 17. V-ATPase overexpression was identified in several diseases, cancer inclusive 18. Targeting V-ATPase in cancer had been reported to promote cancer cell death and repressed oncogenic signals 19, 20. Nevertheless, more mechanistic studies are required to further **e**lucidate how V-ATPase upregulates and promotes anoikis resistance. STAT3 expression was previously correlated with anoikis resistance, aggressive invasion, and migration capability in cancer 21-23. However, whether STAT3 could affect V-ATPase expression in anchorage-independent cells to promote anoikis resistance remains unknown. Herein, we observed that targeting V-ATPase sensitized tumor cells to anoikis via increased ROS production, misfolded protein accumulation, and repressed metastasis *in vivo*. Importantly, STAT3 blockade repressed V-ATPase expression, sensitized tumor cells to anoikis, increased ROS production, and misfolded protein accumulation while STAT3 overexpression upregulated V-ATPase and promoted anoikis resistance.

## Materials and Methods

### Reagent

DMEM, trypsin-EDTA, and Fetal bovine serum (FBS) were purchased from Gibco (CA, USA). Phosphate buffer saline (PBS) and Penicillin-streptomycin (PS) were purchased from HYCLONE (Logan, USA). Chloromethyl-2, 7-dichlorofluorescein diacetate (CM-H_2_DCFDA), and Trizol were from Life Technologies, Invitrogen. Propidium iodide solution was from Biolegend (CA, USA). Stattic, Bafilomycin A, and Concanamycin A were from MedChemExpress (USA). SDS lysis buffer, NP40, and RIPA were from Beyotime (China). BCA and poly-vinylidene fluoride (PVDF) were from Millipore (Ireland). cDNA kit was from TransGen (China). Protease inhibitors and Phosphatase inhibitors were from Roche (Switzerland). Poly-2-hydroxyethyl methacrylate (polyhema), N-acetyl cysteine (NAC), β-actin were from Sigma (USA), HRP-conjugated goat anti-mouse IgG (074-1806) from KPL, and HRP-conjugated goat anti-rabbit IgG (E030120-02) from EARTHOX. ATP6V1B2 and ATP6V0D1 antibodies were from Proteintech (China). STAT3, p-STAT3, and tubulin antibodies were from Cell Signaling Technology (USA). Enhanced chemiluminescence detection kit was from Millipore (USA).

### Cell line

Human cervical cancer (HELA), human breast cancer (MCF-7), and murine melanoma (B16F10) cell lines were obtained from the Chinese Academy of Sciences cell bank and cultured in DMEM supplemented with 10% FBS, 1% PS, in an incubator at 37 °C in a humidified atmosphere containing 5% CO_2_. Cells were cultured for either 3, 6, 24, or 48 hours in commercially available culture dishes to denote adherent condition and in 20 mg/ml polyhema pre-coated dishes (dissolving 2 g in 100 ml 95% ethanol to make 20 mg/ml solution kept in 65 °C water bath for 15 minutes and swirled by rotation for several hours) to denote cells in suspension.

### Plasmid and Transfection

Mammalian Expression vector of Homo sapiens STAT3 (6774, NM_139276.2) with N terminal Flag tag (pCMV-Flag-STAT3) was purchased from Public Protein/Plasmid Library, Geneppl technology, co, Ltd (China). Transfection was performed using Lipofectamine 3000 according to the manufacturer's protocol (Invitrogen).

### RNA-seq and data analysis

Total RNA extracted from HELA cultured in adherent (0 hours) and polyhema pre-coated dishes termed suspension cells (3 and 6 hours) were used for mRNA library preparation. Completed libraries were sequenced on an Illumina HiSeq instrument. Sequencing was carried out using a 2 × 150 bp paired-end (PE) configuration by GENEWIZ, Inc. (Suzhou, China).

### RNA isolation and quantitative real time-polymerase chain reaction (qRT-PCR)

Cells grown in adherent, suspension, or transfected STAT3 cell lines and control were cultured in suspension for 6 hours were used to determine the mRNA expression of targeted genes. Total RNA was extracted with trizol which was used to generate cDNA. Specific primers used for quantitative real-time-PCR assays were synthesized by GENWIZ Corporation. Primers sequences are shown in Table 1, each sample was run in triplicate with three independent experiments.

### Cell morphology imaging

For cell aggregation study, 1x10^5^ cells were seeded in 12-well plates pre-coated with polyhema and treated immediately with or without bafilomycin A (10 nM) or stattic (5 µM). After 24 or 48 hours of culture, cell morphology was observed using an integrated phase-contrast microscope at a magnification of 10x (Olympus CKX53, Tokyo, Japan).

### Cell apoptosis assay

Cells were seeded in triplicates in 12-well plates pre-coated with polyhema at 1x10^5^ cells/well and treated immediately with either bafilomycin A, concanamycin A, stattic, or NAC following specified time points in the figure legends. Also, transfected STAT3 HELA and MCF-7 cells and non-transfected control cells were seeded in triplicates in 12-well plates precoated with polyhema at 1x10^5^ cells/well treated with or without bafilomycin A for indicated time points. Cells were harvested, trypsinized, washed with PBS, and stained with propidium iodide according to the manufacturer's instruction for apoptosis detection via flow cytometry analysis. All samples were analyzed using a flow cytometer (CytoFLEX, BECKMAN COULTER).

### Reactive oxygen species (ROS) detection

Cells were labeled with 5 μM of chloromethyl-2, 7-dichlorofluorescein diacetate (CM-H_2_DCFDA) to determine cellular ROS generated between treated and untreated cells. Cells were incubated for 30 minutes protected from light at 37 °C and washed with PBS before analysis with the flow cytometer.

### Protein extraction and immunoblotting

The whole-cell lysate was prepared by lysing cells grown in adherent or polyhema pre-coated dishes in RIPA supplemented with 1× protease inhibitor and 1× phosphatase inhibitor. Protein concentration was determined by BCA assay following the manufacturer's instruction. Equal concentration of proteins was separated by SDS-PAGE, transferred to a PVDF membrane, and blotted with specific antibodies against ATP6V0D1, ATP6V1B2, STAT3, p-STAT3, actin, and tubulin.

Cells grown in suspension were harvested, washed in PBS, and lysed in NP40 buffer for 30 minutes on ice and subsequently centrifuged at 16,000 g for 15 min at 4 °C. The supernatants were designated as the NP40-soluble (NS) fraction. The protein concentrations in the NS fraction were measured by BCA assay. The pellets were re-suspended in the SDS lysis buffer. SDS-soluble (SS) and the NS fraction were analyzed by Western blot to determine k48-linked polyubiquitinated proteins.

Proteins in the membrane were visualized by an enhanced chemiluminescence detection kit and viewed on Amersham Imager 600 (GE Healthcare).

### Tumor models

All animal procedures were approved by the institutional animal care and use committee (IACUC) of Shenzhen Institutes of Advanced Technology.

Male balb/c nude mice at 6-8 weeks of age were purchased from Guangdong Medical Laboratory Animal Center (Guangzhou, China) and housed in the Shenzhen Institutes of Advanced Technology animal facility under pathogen-free conditions. Mice were intravenously injected with 5x10^5^ cells in 100 μl PBS. Mice were blindly divided into two groups; control and bafilomycin A group (0.1mg/kg IP injection daily). Bafilomycin A treatment commenced on day 2 after tumor injection. The body weights of the mice were recorded every other day.

### Statistical analysis

All analyses were performed using GraphPad Prism Software (San Diego, CA). Data were expressed as mean ± SEM of at least three independent experiments. Student's t-test, one-way, or two-way ANOVA with Tukey's post-test were used for data analysis while *p* values < 0.05 were considered to be significant. In figures, asterisks were used as follows: *, *p* ≤ 0.05; **, *p* ≤ 0.01; ***, *p* ≤ 0.001; and ****, *p* ≤ 0.0001.

## Results

### ECM detachment upregulates V-ATPase expression in tumor cells

**Figure 1:**
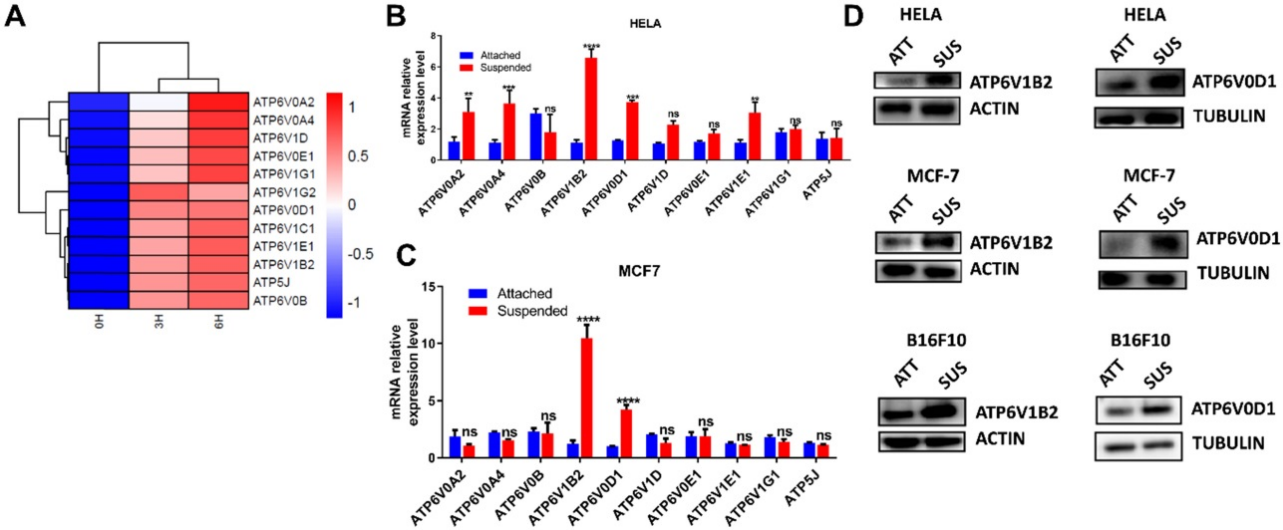
**ECM detachment upregulates V-ATPase expression in tumor cells.** (A) Hierarchical clustering of differentially expressed genes between HELA cell cultured in suspension (3 and 6 hours) and adherent HELA cell obtained after cell passage; (B-C) Relative mRNA levels of some upregulated V-ATPase in HELA (B) and MCF-7 (C) cultured in suspension or adherent conditions for 6 hours. Data represent means ± SEM, ** *p*< 0.01, ***p <0.001, ****p < 0.0001, ns, no significant difference; the experiments were repeated at least three times with similar results. (D) Lysates of HELA, MCF-7, and B16F10 cells cultured in adherent (ATT) and suspension (SUS) conditions for 6 hours were evaluated for ATP6V1B2 and ATP6V0D1 by western blot.

To investigate the molecular changes following ECM detachment, HELA was cultured in a low adherent condition for 3 and 6 hours while adherent cells were collected at 0 hours to serve as the control. Next, transcriptomic analysis was performed to evaluate molecular dynamics which revealed upregulation of V-ATPase in cells cultured in low adherent condition compared to the control (Fig. 1 A).

To ascertain this finding, we investigated most of the upregulated genes by qRT-PCR in HELA and MCF-7. Our result showed consistent upregulation of ATP6V0D1 and ATP6V1B2 in cells grown in suspension compared to adherent cells (Fig. 1B-C). We proceeded to evaluate the protein expression of ATP6V0D1 and ATP6V1B2 in HELA, MCF-7, and B16F10 cells. Similar to RNA-seq and qRT-PCR data, immunoblot analysis further confirmed the upregulation of ATP6V0D1 and ATP6V1B2 in cells cultured in suspension compared to the attached cancer cells (Fig. 1D).

### V-ATPase blockade sensitizes tumor cells to anoikis, increases ROS generation and misfolded protein accumulation

**Figure 2:**
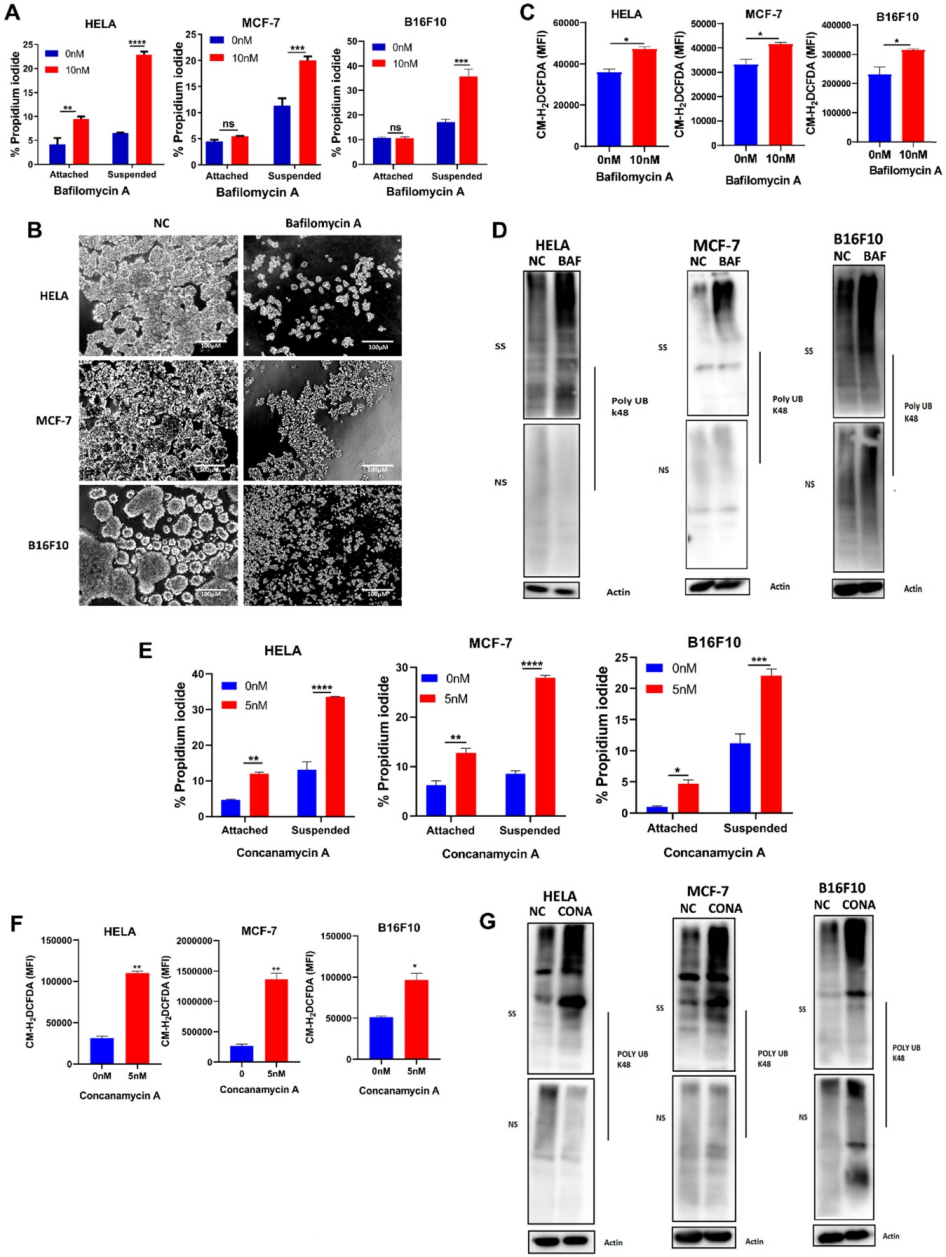
**V-ATPase blockade sensitizes tumor cells to anoikis, increase ROS generation and misfolded protein accumulation.** (A) Percentage apoptosis of HELA, MCF-7, and B16F10 cultured in normal (attached) and polyhema pre-coated (suspended) dishes treated with or without 10 nM bafilomycin A for 24 hours; (B) Phase-contrast microscopic images of HELA, MCF-7, and B16F10 cultured in polyhema pre-coated 12 well plates treated with or without 10 nM bafilomycin for 48 hours, scale bar - 100 µM (C) Mean fluorescence intensity of HELA, MCF-7 and B16F10 treated with or without bafilomycin A (10 nM) for 24 hours and stained with CM-H_2_DCFDA (5 μM) for 30 minutes before analysis with flow cytometer; (D) K48 polyUb-modified proteins in ECM-detached HELA, MCF-7 and B16F10 cells treated with or without bafilomycin A for 24 hours, SS and NS fractions are shown. NS - NP40 soluble fraction, SS - SDS soluble fraction. (E) Percentage apoptosis of HELA, MCF-7, and B16F10 cultured in normal and polyhema pre-coated dishes treated with or without 5 nM concanamycin A for 24 hours. (F) Mean fluorescence intensity of HELA, MCF-7, and B16F10 treated with or without concanamycin A (5 nM) for 24 hours and stained with CM-H2DCFDA (5 μM) for 30 minutes before analysis with a flow cytometer (G) K48 polyUb-modified proteins in suspended HELA, MCF-7 and B16F10 cells treated with or without concanamycin A for 24 hours, SS and NS fractions are shown. NS - NP40 soluble fraction, SS - SDS soluble fraction. Data are expressed as means ± SEM. Representative result from three independent experiments (): *p < 0.05, **p < 0.01 ***p < 0.001 ****p< 0.0001, ns no significant difference.

To investigate the function of V-ATPase in anchorage-independent growth, we treated HELA, MCF-7, and B16F10 cell lines with a specific V-ATPase complex inhibitor - bafilomycin A in (adherent) attached and (polyhema pre-coated) suspended condition. Firstly we were interested in how inhibiting V-ATPase will impact cell death in both culture conditions. Our results showed increased susceptibility of suspended cells to bafilomycin A-induced cell death compared to attached cells (Fig 2A). Next, we examined the morphological changes on inhibition of V-ATPase expression in cells grown in polyhema pre-coated plates. Our data showed that in detached condition, HELA, MCF-7, and B16F10 cells showed large, multicellular aggregates while treatment with bafilomycin A decreased the cellular aggregates (Fig. 2B). These results indicate V-ATPase is required for anoikis evasion in tumor cells.

Studies had reported alteration in reactive oxygen species (ROS) generation following ECM detachment 11, 24, we next probed ROS level via CM-H_2_DCFDA dye following pharmaceutical blockade of V-ATPase. We observed an increase in cellular ROS generation in the bafilomycin A treated groups (Fig. 2C) compared to non-treated for all cancer cells. Considering the susceptibility of cancer cells to anoikis and increased ROS production, we proceeded to examine the impact of V-ATPase inhibition on k48 polyubiquitin as Chen et al., previously reported accumulation of misfolded proteins to be associated with oxidative stress during anchorage-independent growth 25. Interestingly, we observed a significant increase in misfolded protein accumulation in the bafilomycin A treated groups compared to the untreated groups (Fig. 2D). Since V-ATPases are large protein complexes, we employed another specific V-ATPase inhibitor (concanamycin A) to validate targeting V-ATPase was responsible for sensitizing tumor cells to anoikis. Similar to bafilomycin A, we observed susceptibility to anoikis, increased ROS generation, and misfolded protein accumulation in the concanamycin A treated group for all indicated cancer cells cultured in suspension (Figure 2E- 2G). Altogether, these data confirm the role of V-ATPase in preventing tumor cell death in ECM detached condition.

### Scavenging ROS reduced bafilomycin A-induced anoikis and misfolded protein accumulation

**Figure 3:**
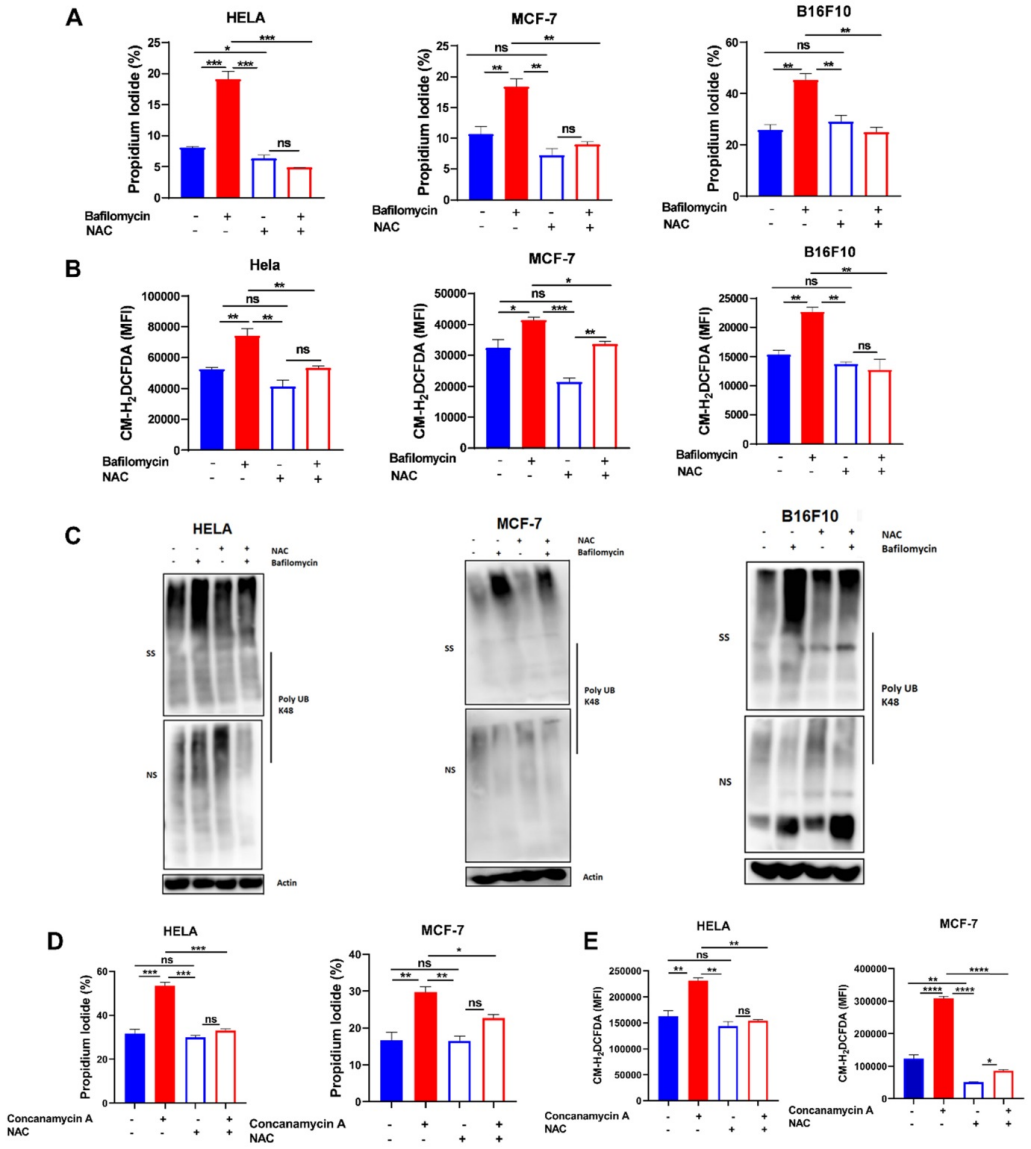
**Scavenging ROS reduced V-ATPase-induced anoikis and misfolded protein accumulation.** (A) Percentage apoptotic HELA, MCF-7, and B16F10 cells treated with or without 10 nM bafilomycin A and 2 mM NAC for 24 hours as determined by flow cytometry; (B) Mean fluorescence intensity of HELA, MCF-7, and B16F10 treated with or without bafilomycin A and NAC for 24 hours, stained with CM-H_2_DCFDA for 30 minutes and evaluated by flow cytometry; (C) K48 polyUb-modified proteins in ECM-detached HELA, MCF-7 and B16F10 cells treated with or without bafilomycin A and NAC for 24 hours; SS and NS fractions are shown. NS - NP40 soluble fraction, SS - SDS soluble fraction (D) Percentage apoptotic HELA and MCF-7 cells treated with or without 5 nM concanamycin A and 2 mM NAC for 24 hours as determined by flow cytometry. (E) Mean fluorescence intensity of HELA and MCF-7 treated with or without concanamycin A and NAC for 24 hours, stained with CM-H_2_DCFDA for 30 minutes, and evaluated by flow cytometry. Data are expressed as means ± SEM. Representative data from three independent experiments (D-E). One way ANOVA: p*<0.05, **p < 0.01, ***p < 0.001, ****p< 0.0001, ns, no significant difference.

To further confirm the role of ROS in V-ATPase inhibition-induced anoikis and misfolded protein accumulation in these tumor cells, we treated cells with 2 mM NAC in the presence or absence of bafilomycin A to evaluate susceptibility to anoikis, ROS generation, and misfolded protein accumulation. As hypothesized, we observed reduced efficacy of bafilomycin A to promote anoikis, ROS generation, and misfolded protein accumulation (Fig. 3A-C). Similarly, treatment of HELA and MCF-7 cells with NAC in the presence or absence of concanamycin A abrogated susceptibility of cancer cells to concanamycin A-induced cell death via impaired ROS generation (Fig. 3D-E). These suggest V- ATPase inhibition sensitizes tumor cells to anoikis through ROS generation.

### STAT3 regulates V-ATPase expression in tumor cells to promote anoikis resistance

**Figure 4:**
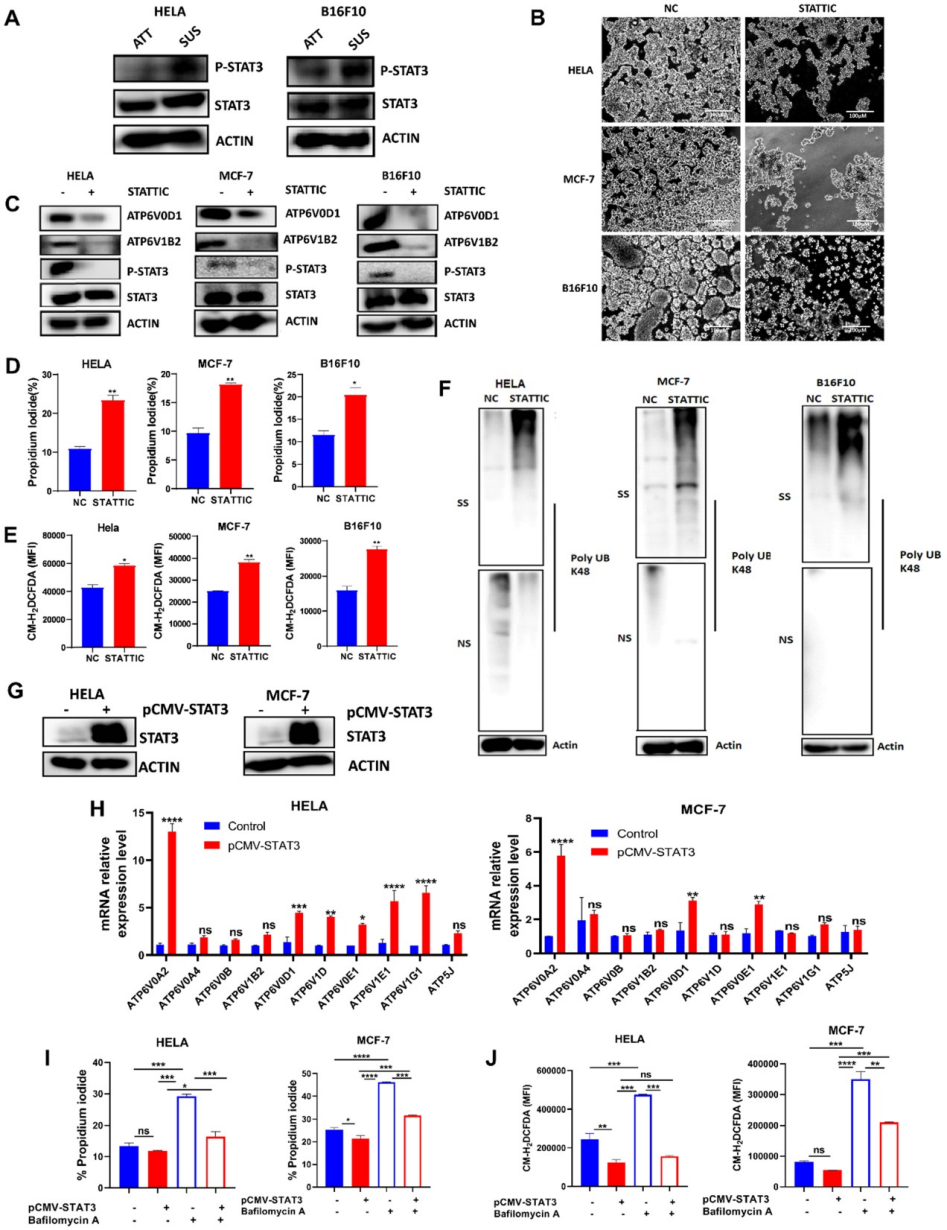
** STAT3 regulates V-ATPase expression in tumor cells to promote anoikis resistance.** (A) Immunoblot analysis for STAT3 and p-STAT3 expression in HELA and B16F10 cells cultured for 6 hours either in adherent and suspension condition (B) Phase-contrast microscopic images of HELA, MCF-7, and B16F10 cells cultured in suspension and treated with or without 5 µM stattic for 24 hours (C) Protein expression of STAT3, p-STAT3, ATP6V0D1, ATP6V1B2 from the total lysate of HELA, MCF-7 and B16F10 cultured in suspension in the presence or absence of 5 µM stattic for 6 hours; (D) Percentage apoptosis of HELA, MCF-7 and B16F10 cells treated with or without STAT3 inhibitor-stattic (5 µM) for 24 hours; (E) Mean fluorescence intensity of indicated cells treated with or without stattic for 24 hours and labeled with CM-H2DCFDA as determined by flow cytometer. (F) K48 polyUb-modified proteins in matrix-detached HELA, MCF-7, and B16F10 cells treated with or without stattic for 24 hours; SS and NS fractions are shown. NS - NP-40 soluble fraction, SS - SDS soluble fraction; (G) Immunoblot analysis for STAT3 expression in HELA and MCF-7 cells transfected with pCMV-FLAG-STAT3 cultured in suspension for 6 hours. (H) Relative mRNA levels of some V-ATPase genes in HELA and MCF-7 expressing pCMV-STAT3 cultured for 6 hours. (I) Percentage apoptotic HELA and MCF-7 expressing pCMV-STAT3 cells treated with or without 10 nM bafilomycin A for 24 hours as determined by flow cytometry. (J) Mean fluorescence intensity of HELA and MCF-7 expressing pCMV-STAT3 with or without bafilomycin A treatment for 24 hours, stained with CM-H2DCFDA for 30 minutes, and evaluated by flow cytometry. Data are expressed as means ± SEM. Representative data from three independent experiments (H-J). One way ANOVA: p*<0.05, **p < 0.01, ***p < 0.001, ****p< 0.0001, ns, no significant difference.

STAT3 had been reported to play a crucial role in suppressing ROS signaling to favor tumorigenesis 26, 27. Also, STAT3 activation had been reported in anchorage-independent cancer cells 21-23, 28. Therefore, we determined protein expression of STAT3 and p-STAT3 in HELA and B16F10 cells grown in attached and detached conditions. Immunoblot analysis showed that cells grown in anchorage-independent conditions induced the activation of p-STAT3 in HELA and B16F10 cells compared to the attached condition (Fig. 4A). Moving forward, we investigated how the blockade of STAT3 activity using small molecule inhibitor - stattic will impact the morphology of HELA, MCF-7, and B16F10 cells when grown in polyhema pre-coated culture plates. Interestingly, we observed reduced cellular aggregates on treatment with STAT3 inhibitor compared to the untreated cells (Fig. 4B). Then we asked if STAT3 signaling affects V-ATPase expression observed in these cancer cells grown in anchorage-independent conditions. Immunoblot data confirmed the ability of stattic to repress the phosphorylation of p-STAT3 (Fig. 4C). Importantly, blocking STAT3 signaling in cancer cells inhibited the expression of two members of the V-ATPase family - ATP6V1B2 and ATP6V0D1 (Fig. 4C). Furthermore, we evaluated the effect of stattic on anoikis, ROS generation, and misfolded protein accumulation. Our data showed that stattic promoted anoikis (Fig. 4D), increased ROS production (Fig. 4E), and induced misfolded protein accumulation (Fig. 4F) in HELA, MCF-7, and B16F10. To validate STAT3 involvement in regulating V-ATPase expression, we transiently overexpressed STAT3 in HELA and MCF-7 and confirmed with immunoblot analysis (Fig. 4G). Next, we investigated the mRNA expression level of V-ATPase genes upregulated in suspended cells shown in Fig 1A. Interestingly, we observed upregulation in mRNA expression of some members of the V-ATPase family in STAT3 overexpressed tumor cells (Fig 4H). Furthermore, we investigated the effect of V-ATPase inhibition with bafilomycin A on STAT3 overexpressed cancer cells. As expected, STAT3 overexpression protected cells from bafilomycin A-induced anoikis and impaired ROS generation (Fig 4I-J). These suggest an unreported role of STAT3 in regulating V-ATPase expression in anchorage-independent cells - a precondition for metastasizing tumor cells.

### V-ATPase inhibition impaired pulmonary metastasis *in vivo*

**Figure 5:**
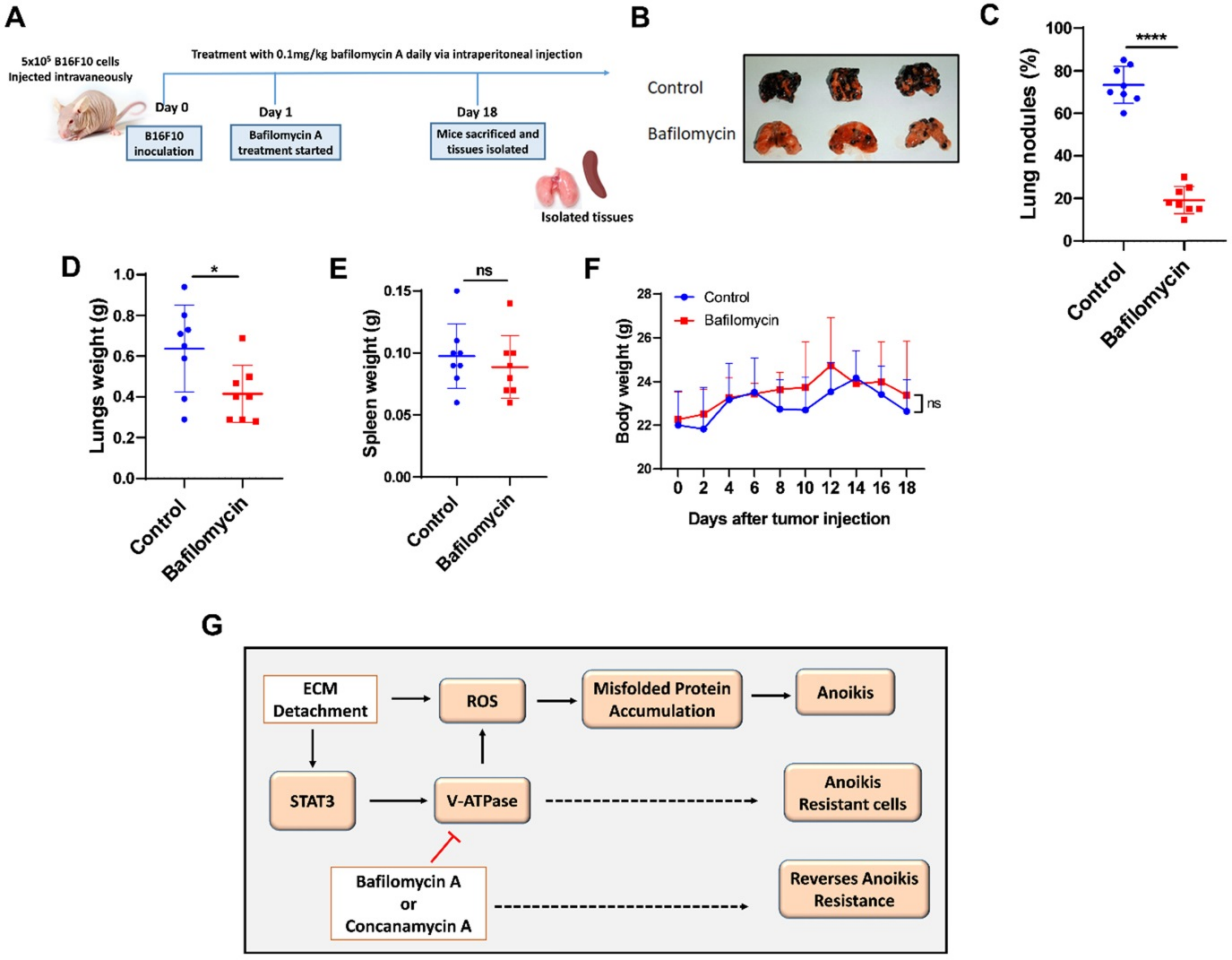
** V-ATPase inhibition impaired pulmonary metastasis *in vivo*.** (A) Experimental design for male balbc/nude mice injected intravenously with 5x10^5^ B16F10 for 18 days; treatment with bafilomycin A daily commenced the following day after tumor injection; (B) Images of lungs, (C) Percentage of lung tumor nodules; (D) Lungs weight kinetics; (E) Spleen weight kinetics; (F) body weight kinetics of mice treated with bafilomycin A daily on day 18 post tumor inoculation; (G) Proposed study model; Results are means ± SEM pooled from two independent experiments using unpaired student t-test: *p <0.05, ****p < 0.0001, ns, no significant difference.

To confirm these results *in vivo*, we examined the effect of V-ATPase blockade on metastasis in melanoma B16F10-balb/c nude mice treated daily with 0.1 mg/kg of bafilomycin A for 17 days compared to the control mice (Fig. 5A). Our study showed that bafilomycin A treated mice had reduced metastatic potential, lung nodules formation, and lung weight compared to the control mice (Fig. 5B-D). Notably, treatment with bafilomycin A did not alter body nor spleen weight compared to the control group (Fig. 5E and F), suggesting its safety in being used to prevent metastasis.

## Discussion

Anoikis resistance is a common hallmark of metastatic cancer cells that can be exploited to prevent metastasis 6. However, the molecular mechanisms remain to be profoundly understood. V-ATPase has been shown to promote tumorigenicity via enhanced acidification of the extracellular environment thus aiding structural and biochemical transformations conferring flexibility to cancer cells for metastasis 20, 29. In this study, we observe the overexpression of V-ATPase in RNA-sequencing data from HELA cells cultured in ECM detached condition which was further confirmed with qRT-PCR and western blot analysis in human breast cancer (MCF-7) and murine melanoma (B16F10) cells. Next, we demonstrated that V-ATPase inhibition in cancer cells promotes anoikis and impaired tumor metastasis. Mechanistically, we identified STAT3 activation to be critical for the upregulation of V-ATPase, thus pharmacological blockade of STAT3 repressed V-ATPase expression leading to anoikis via ROS-induced misfolded protein accumulation (Fig. 5G). Our results reveal a more detailed axis that drives anoikis resistance and subsequent metastasis, which provides targets and guidance for metastasis prevention.

Blocking V-ATPase via its specific inhibitors bafilomycin A and concanamycin A, increased ROS production thereby enhanced tumor cells' susceptibility to anoikis. This is in agreement with a previous study that utilized archazolid, another V-ATPase inhibitor that increased sensitivity to anoikis 19. Chen et al. previously reported clearance of misfolded protein by cancer cells as an underlying mechanism by which tumorigenesis is promoted 25. Also, this team established a relationship between increased ROS and misfolded protein accumulation which inhibited oncogenic growth. Importantly, both inhibitors used in our study demonstrate increased ROS production and led to misfolded protein accumulation during anchorage-independent growth. Our recent findings further validate accumulation of misfolded protein is cytotoxic as earlier reported by Brohem et al., 30.

Oxidative stress has been implicated in the impairment of the ubiquitin-proteasome system thus leading to misfolded protein accumulation which had been reported to inhibit oncogenic phenotypes initiation, transformation, and aggression of tumor 30, 31. N-acetyl cysteine (NAC) is an established ROS scavenger 32. To clarify the role of increased ROS production following bafilomycin A and concanamycin A treatment in our study as well as their therapeutic potential in sensitizing cells to anoikis; NAC was used to treat these cancer cells in the presence or absence of bafilomycin A and concanamycin A. Our data showed reduced sensitivity to anoikis, reduced ROS generation, and clearance of misfolded protein in all cancer cells co-treated with bafilomycin A and NAC which further corroborate previous studies on antioxidant activity promoted anoikis resistance 14, 16, 33. On the contrary, Schempp *et al.*, reported that archazolid-induced ROS and BIM degradation in T24 urinary cells may be a mechanism explored by detached cells to promote anoikis resistance. This was because co-treatment of archazolid and NAC demonstrated increased susceptibility of T24 cells to anoikis compared to archazolid treated cells alone 19. This varying observation with different V-ATPase inhibitors or cancer cells suggests new studies to fully understand the exact mode of action of these inhibitors for informed use in metastasizing tumor cells.

Previous studies reported STAT3 activity favors anoikis resistance and metastasis in cancer 21-23, 28, however, the role of STAT3 in mediating anoikis resistance and oncogenic phenotypes in cancer is yet to be fully understood. Here we report that phosphorylation of STAT3 regulates V-ATPase expression in cancer cells thus promote anoikis resistance. Interestingly, we found that inhibition of STAT3 signaling represses V-ATPase expression and sensitizes cells to anoikis. On the other hand, cancer cells overexpressing STAT3 demonstrated increased expression of V-ATPase, reduced sensitivity to bafilomycin A treatment, impaired ROS generation, and facilitated anoikis resistance. Importantly, this study demonstrates for the first time that STAT3 modulates V-ATPase activity to confer anoikis resistance and metastasis as treatment with stattic showed similar effects as V-ATPase inhibition.

The limitation of this study is that since the V-ATPase family is multi-complex, it is difficult to genetically deplete a particular member of the V-ATPase to specifically study their role(s) in cancer cells. Hence, we explored two small molecules - bafilomycin A and concanamycin A, known specific V-ATPase inhibitors 34. Our data revealed both bafilomycin A and concanamycin A sensitized cancer cells to anoikis, which not only confirmed the involvement of V-ATPase in anoikis resistance but also provides two candidate inhibitors against cancer metastasis. Therefore, more studies are required to elucidate other potential mediators of V-ATPase upregulation in ECM detached cancer cells and how various V-ATPase inhibitors induce anoikis or counter mechanisms aiding anoikis resistance will provide deeper insight to target metastasis.

In summary, this study identified V-ATPase as a targetable protein vital for anoikis resistance based on objective screening by global profile gene expression alterations in forced suspension culture and elucidated STAT3 as a key regulator of V-ATPase expression in anchorage-independent cells; thus enhance tumor cells to be anoikis resistant due to impaired ROS production. Targeting the STAT3/V-ATPase axis may be a promising strategy for preventing cancer metastasis.

## Figures and Tables

**Table 1:** Primer sequences.

S/N	Vacuolar ATPase given name	Forward- F and Reverse- R (5'-3')
1	ATP6V0A4	F- AGCCTGGGAGGAGAAGGAG R- CCTCGGTCCCAGCTTCCT
2	ATP6V1B2	F- CAGCCTCGCCTCACATACAA R- GTGCCATCCGGTAAGGTCAA
3	ATP6V0D1	F- GTCGTTCTTCCCGGAGCTTT R- TTCAAGTCCTCTAGCGTCTCG
4	ATP6V0E1	F- GCTCAGTCTTTGAGGTCACGA R- TTTGTGGAGTCGGCACAGTT
5	ATP6V1G1	F- CTTGCTCTCAGAATCGCTGC R- CGGTTCTTTCTTTTGCGGGC
6	ATP5J	F- GGACTGAGTGCAAGAATCAGC R- ACACCAATGTTCCTCCGCAA
7	ATP6V1E1	F- GAAGAAATAGATGCAAAGGCAGAAG R- ACCACCTTGCTGAGTCTCTGT
8	ATP6V0A2	F- CACAGGGCAGGAGTATGTCC R- GGTTCACCCCGAAGCAACTA
9	ATP6V0B	F- GCTAGCACTGCTCTACTCCG R- TCAGGAACCATGCCACATCA
10	ATP6VID	F- GCTGAAGCCAAGTTCACAGC R- ACTGGCAAAGTAACACCTGCT
11	β-Actin	F- ATTGGCAATGAGCGGTTCCG R- AGGGCAGTGATCTCCTTCTG
